# Parabiosis, Assembloids, Organoids (PAO)

**DOI:** 10.1002/advs.202511671

**Published:** 2025-09-27

**Authors:** Yang Hong, Lavonda Li, Lijie Yan, Long Bai, Jiacan Su, Xingcai Zhang

**Affiliations:** ^1^ Institute of Translational Medicine Shanghai University Shanghai 200444 China; ^2^ MedEng‐X Institutes Shanghai University Shanghai 200444 China; ^3^ National Center for Translational Medicine (Shanghai) SHU Branch Shanghai University Shanghai 200444 China; ^4^ School of Medicine Shanghai University Shanghai 200444 China; ^5^ World Tea Organization Cambridge MA 02139 USA; ^6^ School of Engineering Stanford University Stanford CA 94305 USA; ^7^ Department of Medicine Stanford University Stanford CA 94305 USA; ^8^ Wenzhou Institute of Shanghai University Wenzhou 325000 China; ^9^ Department of Orthopedics Xinhua Hospital Affiliated to Shanghai Jiao Tong University School of Medicine Shanghai 200092 China

**Keywords:** artificial intelligence (AI), CRISPR, organ‐on‐a‐chip, parabiosis assembloids organoids (PAO), precision medicine

## Abstract

The research and treatment of major diseases challenge global public health, necessitating advanced disease models. Existing approaches have clear limitations: two‐dimensional cell cultures lack multi‐organ interactions, clinical trials are costly and ethically constrained, and animal models, focused on single organs, fail to replicate systemic regulation. Parabiosis, which connects two organisms via shared circulation, provides insights into systemic factors and multi‐organ interactions but has limited applicability to humans. Furthermore, organoids are three‐dimensional structures formed through stem cell self‐organization that replicate the functions of individual tissues and advance personalized medicine; however, they cannot model inter‐tissue interactions. Assembloids overcome these constraints by integrating diverse organoids, enabling sophisticated simulation of multi‐organ dynamics. The integration of these parabiosis, assembloids, organoids (PAO) models with emerging technologies, such as artificial intelligence for precision analytics, CRISPR‐based gene editing for disease mechanism elucidation, organ‐on‐a‐chip platforms for dynamic environmental control, and soft robotics for replicating physiological biomechanics, promises to revolutionize disease modeling, regenerative medicine, and precision therapeutics. This review evaluates parabiosis, assembloids, and organoids, highlighting their development, current limitations, and transformative potential when combined with frontier biomedical engineering approaches to address complex human diseases.

## Graphical abstract

This review evaluates parabiosis, organoids, and assembloids as complementary disease models spanning systemic, organ, and multi‐organ levels. It highlights their construction strategies, applications, and current limitations, while emphasizing their integration with frontier technologies such as artificial intelligence, organ‐on‐a‐chip, CRISPR, and soft robotics to advance disease modeling, regenerative medicine, and precision therapeutics.

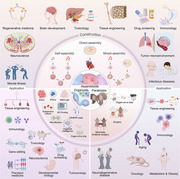

## 1 Introduction

The research and treatment of major diseases such as cancer, neurodegenerative disorders, cardiovascular diseases, and infectious diseases present significant challenges to global public health. These complex conditions require multi‐level research models spanning molecular, cellular, and systemic scales. However, existing models exhibit critical limitations in addressing complex biological processes. Two‐dimensional (2D) cell cultures offer high experimental controllability and suitability for high‐throughput screening but fail to replicate the structural complexity and multi‐organ interactions necessary to fully model disease progression. While clinical trials provide direct validation in human patients, their high costs, lengthy timelines, and ethical constraints limit widespread early‐stage application. Bridging the gap between 2D cultures and clinical studies, animal models have been widely employed in scientific research to simulate systemic pathological processes. These models have proven valuable in basic research and drug development but are typically designed to focus on single‐organ or localized pathological processes. As a result, they struggle to accurately reproduce the intricate multi‐organ interactions and systemic regulatory mechanisms characteristic of complex diseases, highlighting their limitations in comprehensively modeling such conditions.

The parabiosis model, a specialized animal experimental technique, offers a unique perspective for studying systemic regulation and inter‐organ interactions. By surgically connecting two animals to share a circulatory system, parabiosis enables direct observation of systemic factors’ effects on multiple tissues and organs. Compared to traditional animal models, parabiosis reveals cross‐organ physiological interactions and provides a platform to investigate the role of systemic regulatory factors in disease progression, offering insights more aligned with human physiology. This technique dates back to 1864, when Paul Bert first described it. Since then, parabiosis has been widely applied in fields such as endocrinology, immunology, and aging research, significantly impacting stem cell biology and regenerative medicine. Through heterochronic parabiosis, where young and old animals are connected, researchers have found that factors in the blood of young animals can partially reverse aging effects in older animals.^[^ ^1^ ^]^ Parabiosis can reveal complex biological processes at the level of real organ interactions, particularly concerning systemic diseases, metabolic regulation, and immune system function. However, the physiological differences between species limit the translational relevance of parabiosis findings to humans. Significant disparities in immune responses, metabolic pathways, and organ structures between animal models and humans often impede the direct application of results. For example, mice differ from humans in tumor progression, drug responses, and physiological mechanisms, leading to discrepancies in therapeutic efficacy and side effects observed in animal studies. While parabiosis introduces a novel approach to studying cross‐organ interactions, its clinical applicability remains constrained by interspecies differences, highlighting the urgent need for physiologically relevant models more representative of human biology.

**Figure 1:**
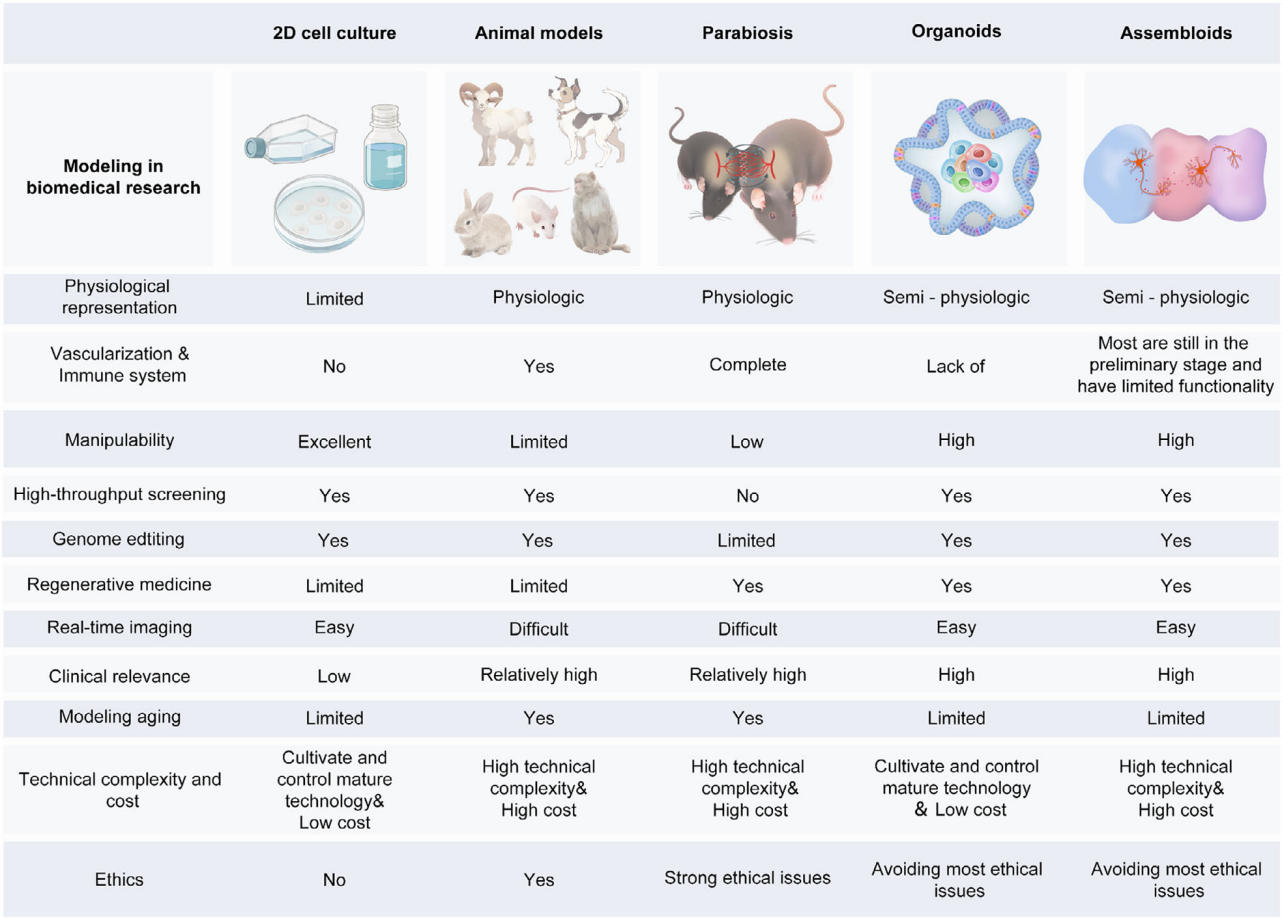
Comparison of different biomedical research models.

With the cancellation of mandatory animal testing requirements in the United States in 2022, the importance of in vitro models that simulate complex tissue behavior in disease research has become increasingly evident. Organoids have thus emerged, formed through the self‐organization of stem cells under three‐dimensional (3D) culture conditions to create functional tissue complexes. In cancer research, patient‐derived tumor organoids effectively replicate the tumor microenvironment, enabling the study of tumor‐stroma interactions, heterogeneity, and drug resistance.^[^ ^2^ ^]^ Colorectal cancer organoids have successfully simulated genetic diversity and treatment responses, highlighting their potential in personalized therapy.^[^ ^3^ ^]^ However, organoids typically model only single tissues and cannot reproduce the complex interactions between multiple tissues or organs. Consequently, assembloids have gained attention in recent years. Assembloids are complex structures that self‐assemble through multicellular systems, capable of simulating interactions between various tissues. Brain assembloids have played a crucial role in advancing our understanding of neural circuits and interactions between different brain regions.^[^ ^4^ ^]^ Assembloids demonstrate broad potential applications in tissue engineering, organ regeneration, and complex disease modeling, promising to address the limitations of organoids in simulating inter‐tissue interactions and system‐level behaviors, thereby further promoting the development of precision medicine and personalized treatment (**Figure** **1**). These conceptual relationships illustrate distinct modes of connection. In the parallel view, each model provides independent but complementary insights. In the nested view, organoids and assembloids can be positioned within parabiosis to examine systemic influences on specific tissues. In the integrated view, the three models act together, linking organismal, organ, and multi‐tissue levels.

In summary, the three models—parabiosis, assembloids, and organoids—form a hierarchical framework that spans from systemic to organ and multi‐organ levels. Parabiosis reveals circulating factors and whole‐body regulation, organoids reconstruct tissue‐specific functions under controlled conditions, and assembloids integrate multiple tissues to capture higher‐order interactions. Each model has unique advantages and complements the others, yet systematic reviews in this field remain scarce. Therefore, we first provide an overview of each model's definition, historical development, construction strategies, and application scenarios, while also addressing their challenges and future prospects. Building on this foundation, we conduct an in‐depth comparison across dimensions such as physiological relevance, vascularization and immune system integration, technical complexity and cost, regenerative medicine, and ethical considerations. This systematic analysis highlights their strengths and limitations in bridging “in vivo‐in vitro” processes and underscores their potential application value. Future integration of these three models is expected to drive precision medicine and enable new advances in the study of complex diseases.

## 2 Parabiosis

### 2.1 Development History

Parabiosis has emerged as an important experimental paradigm in aging, immunology, and regenerative medicine. Introduced in 1864 by Paul Bert, parabiosis initially involved surgically connecting animals’ circulatory systems to study physiological exchanges and systemic effects.^[^ ^5^ ^]^ The term “parabiosis” was formally coined by Sauerbruch and Heyde in 1908, describing artificially induced vascular sharing across species.

Significant advancements occurred in the mid‐20th century when McCay et al. demonstrated rejuvenation effects in old mice connected to young counterparts, revealing the influence of circulating blood factors on aging processes. Subsequent studies, such as those by Halberg and colleagues, employed parabiosis to investigate immune tolerance and transplant rejection, significantly advancing immunological understanding and transplantation medicine.

**Figure 2:**
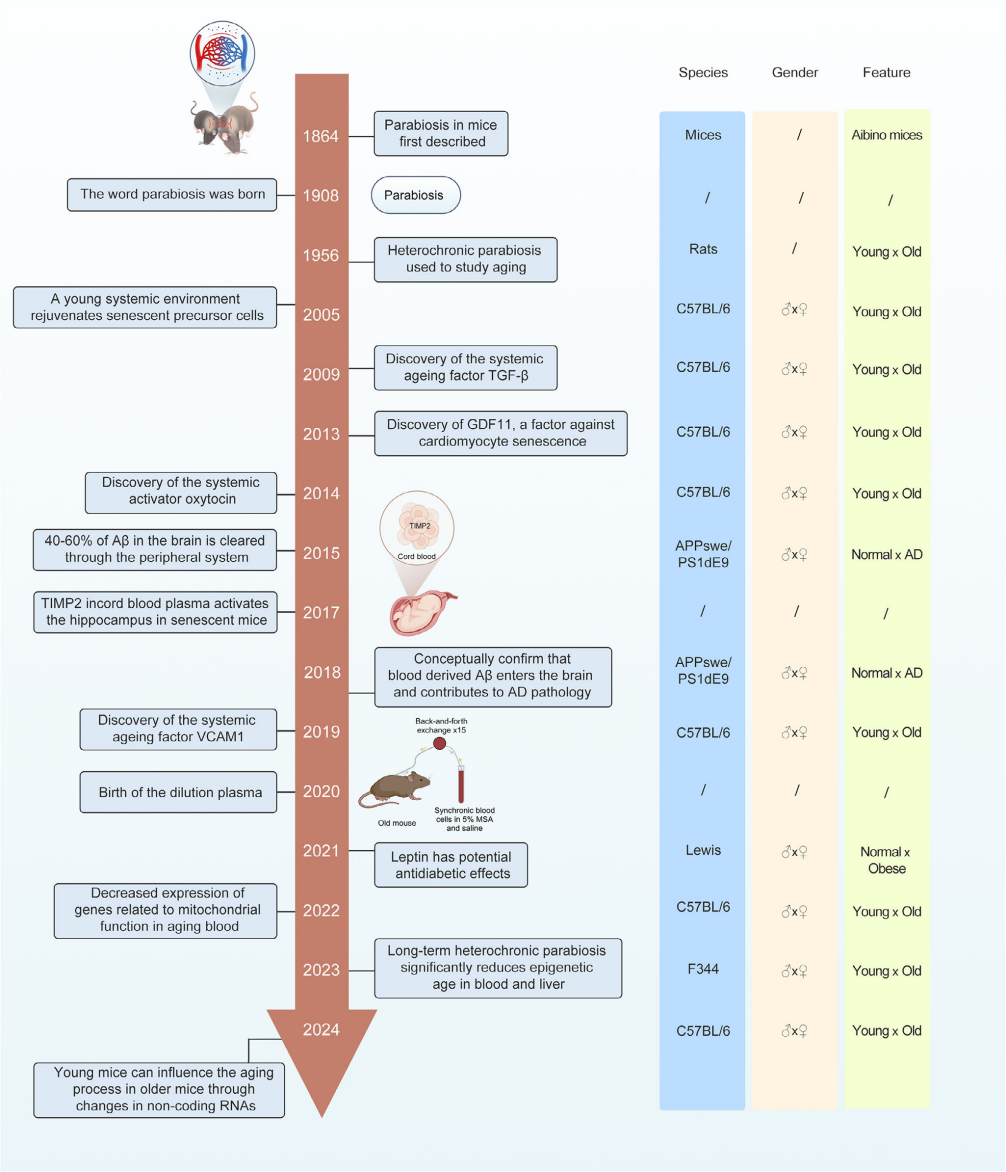
A brief history of parabiosis.

Modern parabiosis studies, notably by Conboy and Wagers groups, have identified critical systemic aging regulators including TGF‐β, oxytocin, and GDF11, linking these factors to enhanced tissue regeneration and age reversal.^[^ ^1^, ^6^ ^]^ These findings extended parabiosis research to neurodegenerative diseases, such as Alzheimer's and Parkinson's. Nevertheless, clinical translation remains controversial; despite initial commercialization efforts, such as Ambrosia's plasma infusions in 2017, the FDA warned in 2019 of inadequate validation and potential risks. Recent advances propose plasma dilution as an alternative strategy, showing promising anti‐aging and therapeutic potential in animal models of neurodegeneration (**Figure** **2**).

Current research continues exploring parabiosis‐inspired strategies for personalized medicine, particularly focusing on delaying aging, promoting regeneration, and managing neurodegenerative diseases, though clinical application remains nascent.

### 2.2 Construction Strategies

**Figure 3:**
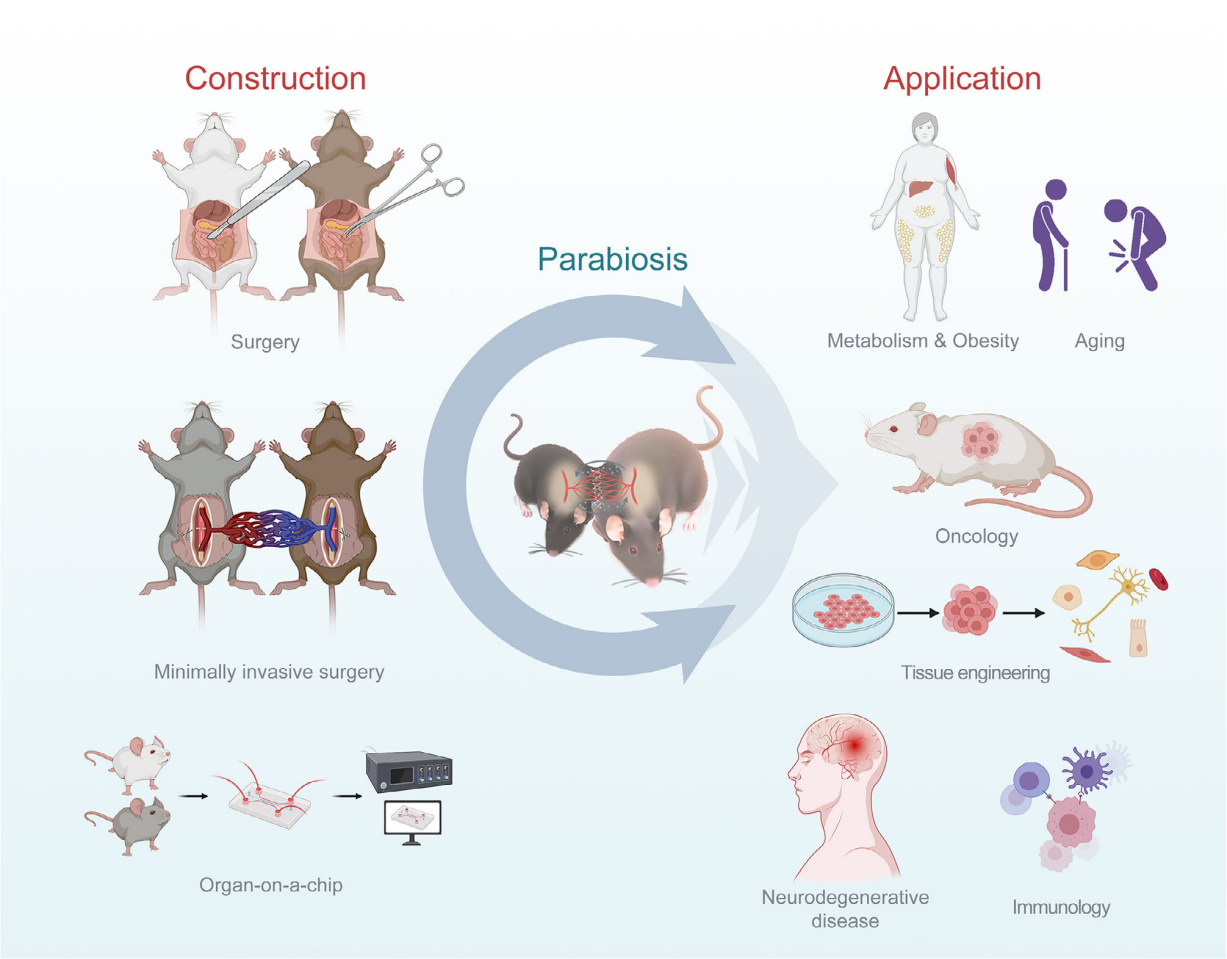
Construction and application of parabiosis.

The construction strategies for parabiosis have evolved from basic vascular connections to more complex and precise methods, including minimally invasive surgery, molecular labeling, gene editing, and integration with organ‐on‐a‐chip systems (**Figure** **3**).

#### 2.2.1 Basic Surgical Connections and Heterochronic Parabiosis

Surgical anastomosis is the earliest approach to establishing parabiosis, involving the physical connection of two organisms through the surgical joining of their skin, muscle, and vascular systems. Originating from Paul Bert's experiments in the 19th century, this method enabled early investigations into the systemic physiological effects of shared blood circulation. Historically, this approach facilitated fundamental studies on metabolism, aging, and other systemic physiological processes.

A notable variation, heterochronic parabiosis, specifically connects young and aged individuals to assess the impact of age‐related circulating factors. Initially explored systematically by McCay et al. in 1956, this technique demonstrated rejuvenating effects of young blood on the tissues and lifespan of older animals. Subsequent research has shown that young blood exposure enhances regeneration by activating stem cell populations across various aged tissues, including skeletal muscle, bone, and nervous system.^[^ ^7^ ^]^

In aged brains, heterochronic parabiosis induces transcriptional reprogramming across neurons, astrocytes, and microglia, reversing age‐associated gene expression patterns. These alterations improve neural stem cell function, attenuate inflammation, and promote synaptic plasticity, learning, and memory. In addition, critical aging‐related signaling pathways, such as Wnt, mTOR, and mitochondrial metabolic networks, are modulated by factors derived from young blood, collectively contributing to tissue rejuvenation.^[^ ^8^ ^]^

#### 2.2.2 Minimally Invasive Surgery and Improved Vascular Connections

Reducing the invasiveness of parabiosis procedures has markedly improved experimental outcomes by decreasing trauma and enhancing animal welfare.^[^ ^9^ ^]^ As early as 1933, Bunster and Meyer modified traditional parabiosis methods by connecting animals at the scapular joint, thoracic cavity, and skin. This adjustment effectively reduced tissue tension, postoperative complications, and mortality rates. Subsequent advancements in surgical instrumentation facilitated the adoption of smaller incisions, precise suturing methods, and rigorous aseptic techniques, further minimizing surgical trauma. Enhancements in anesthetic practices and intraoperative temperature management have also been crucial, significantly reducing physiological stress and improving postoperative recovery.^[^ ^10^ ^]^

#### 2.2.3 Integration of Parabiosis with Organ‐on‐a‐Chip Technology

Combining parabiosis with organ‐on‐a‐chip technology provides precise, high‐throughput in vitro platforms to model heterochronic parabiosis. Such integrated systems allow controlled simulation of cellular communication between young and aged tissues, facilitating the identification of rejuvenating factors involved in tissue regeneration, stem cell activation, and modulation of aging‐related signaling pathways.^[^ ^11^ ^]^ In addition, integrating advanced biosensors within these chips enables sensitive, real‐time detection of circulating biomarkers.^[^ ^12^ ^]^ Collectively, these innovations significantly enhance the resolution and reliability of parabiosis studies, providing deeper insights into aging mechanisms and potential therapeutic targets.

### 2.3 Application Scenarios

Parabiosis, since its establishment, has been extensively utilized in biomedical research, notably in aging, regenerative medicine, immunology, metabolism, neurodegenerative diseases, and oncology. Ongoing integration with advanced techniques, including microphysiological systems and molecular editing tools, promises further transformative impacts on precision and personalized medicine (Figure 3).

#### 2.3.1 Aging, Regenerative Medicine, and Stem Cells

Parabiosis studies have uncovered rejuvenating effects of systemic factors from young animals, notably identifying GDF11 as a pivotal anti‐aging molecule capable of reversing cardiac hypertrophy and promoting vascular and neural regeneration.^[^ ^1^, ^13^ ^]^ In addition, young systemic factors enhance regenerative capacities within the central nervous system by activating oligodendrocyte precursor cells and promoting myelin repair through the recruitment of young‐derived monocytes, demonstrating significant therapeutic potential for aging‐related tissue decline.^[^ ^14^ ^]^

#### 2.3.2 Immunological Research

Studies employing parabiosis have significantly advanced our understanding of immune regulation, offering critical insights into immune tolerance mechanisms, leukocyte migration, and the systemic interplay of immune cells within different tissue environments.^[^ ^7^ ^]^ For instance, transferring immune cells via parabiosis from wild‐type into hepatitis B virus (HBV)‐tolerant mice disrupts established immune tolerance, stimulating antiviral responses. Likewise, parabiosis models revealed that circulating antigen‐presenting cells could trigger autoimmune uveitis in previously healthy tissues, highlighting the systemic nature of autoimmune responses. Furthermore, heterochronic parabiosis demonstrated bidirectional modulation of immune aging, enhancing immune functionality and anti‐tumor immunity in aged animals exposed to young blood.

#### 2.3.3 Neurodegenerative Diseases

Young systemic factors in parabiosis improve neural function and delay neurodegeneration. Young blood factors have been shown to mitigate age‐associated cognitive decline by restoring synaptic plasticity impaired by factors such as β2‐microglobulin (β2M).^[^ ^15^ ^]^ Circulating rejuvenating molecules enhance neural stem cell proliferation, improve neuronal survival, and reinforce blood‐brain barrier integrity.^[^ ^16^ ^]^ Importantly, circulating factors also modulate brain inflammation and metabolism, revealing promising targets for therapeutic intervention in neurodegenerative disorders.

#### 2.3.4 Tumor Research

Parabiosis models have facilitated detailed exploration of immune dynamics within tumor microenvironments.^[^ ^17^ ^]^ Experiments connecting tumor‐bearing animals with healthy counterparts indicate immune cell transfer significantly enhances tumor‐infiltrating CD4+ and CD8+ T‐cell populations, leading to suppressed tumor progression. In addition, parabiosis clarified that tumor‐associated macrophages predominantly originate from circulating monocyte precursors rather than local resident cells.

#### 2.3.5 Metabolism and Obesity Research

Exposure of obese diabetic mice to healthy circulation significantly reverses cardiac hypertrophy, highlighting systemic factors like GDF11 as key regulators. Moreover, leptin transfer via shared circulation effectively restores insulin sensitivity and glucose homeostasis in diabetic models, underscoring systemic endocrine control in metabolic disease management.^[^ ^18^ ^]^

### 2.4 Challenges

#### 2.4.1 Ethical Considerations and Animal Welfare

Parabiosis, while experiencing renewed interest due to its scientific promise, raises critical ethical and animal welfare concerns. Surgical connection of animals imposes significant physiological and psychological stress, highlighting the need for stringent ethical evaluation. Systemic responses observed in mice may not replicate in humans due to divergent cellular targets and pathways. Moreover, cross‐species blood sharing poses challenges such as immune incompatibility, altered pharmacodynamics, and epigenetic divergence. These limitations underscore the need for caution in extrapolation.

In addition, translating parabiosis‐derived rejuvenation concepts into human applications brings profound ethical challenges, such as equitable access and resource allocation. Young‐blood transfusions could disproportionately benefit affluent populations, exacerbating societal inequalities and raising potential risks of exploitation or coercion of blood donors. Therefore, ethical oversight and robust regulations are imperative for responsible development and application of parabiosis technologies.

#### 2.4.2 Reproducibility and Standardization

Despite substantial research advances, parabiosis experiments still face challenges regarding reproducibility and methodological standardization. Variables including surgical precision, timing of vascular connections, animal health status, and environmental factors significantly influence experimental outcomes.^[^ ^9^ ^]^ Moreover, current heterochronic parabiosis models can be complicated by unintended organ sharing or inconsistent circulatory interactions.

#### 2.4.3 Long‐Term Effects and Chronic Responses

Long‐term experiments have shown shortened lifespan and signs of premature aging in younger animals, suggesting that extended exposure to aged systemic environments may outweigh regenerative benefits.^[^ ^19^ ^]^ Moreover, older blood may exacerbate disease progression in younger organisms, potentially promoting chronic inflammation and immune dysregulation. Continuous heterologous blood exchange could thus trigger maladaptive immune responses, tissue dysfunction, and unknown long‐term risks, underscoring the need for cautious evaluation of parabiosis in chronic experimental settings.

#### 2.4.4 Complexity of Systemic Factors and Identification Challenges

A central goal of parabiosis is the identification of circulating factors that influence aging and disease; however, blood harbors a highly complex repertoire of proteins, cytokines, and metabolites, which often act in concert through context‐dependent and time‐sensitive mechanisms. Disentangling individual factor contributions, elucidating causal relationships, and validating their function in specific tissues remain technically demanding.^[^ ^13^, ^20^ ^]^ In addition, spatial heterogeneity and temporal fluctuations in factor concentrations complicate reproducibility and mechanistic interpretation. In practice, candidate systemic factors in parabiosis are profiled through plasma proteomics and immunoassays such as aptamer‐based arrays, proximity extension assays, enzyme linked immunosorbent assay, and luminex, which enable sensitive detection of circulating cytokines and SASP proteins. Transcriptomic analyses, including bulk and single‐cell sequencing, further connect these circulating signals to cellular responses in specific tissues, with the SenMayo gene set serving as a standardized marker of senescence burden.^[^ ^21^ ^]^ Developing robust analytical frameworks and high‐resolution detection tools remains critical to translating these findings into actionable therapeutic strategies.

Parabiosis has revealed the profound influence of systemic factors on aging, regeneration, and disease progression, yet its whole‐body nature limits precise attribution to specific tissues. To dissect these circulating influences in a controlled manner, in vitro platforms that capture organ‐level biology are required. Organoids, generated through stem cell self‐organization under 3D culture conditions, provide a tractable system to validate candidate factors, investigate tissue‐specific mechanisms, and translate systemic insights into organ‐focused models.

## 3 Organoids

### 3.1 Development History

Organoids are miniature, simplified structures of organs formed from stem cells or somatic cells under 3D culture conditions. They can simulate the functions and structures of organs in vitro, making them widely applicable in fields such as disease research, drug screening, and regenerative medicine.^[^ ^22^ ^]^ This concept dates back to early cell culture research in the 20th century, and with the rise of 3D culture techniques, organoid research has gradually evolved into an independent field. In 2009, Hans Clevers first cultured intestinal organoids from intestinal stem cells in vitro, leading to the rapid emergence of organoids as core tools in biomedical research.^[^ ^23^ ^]^

**Figure 4:**
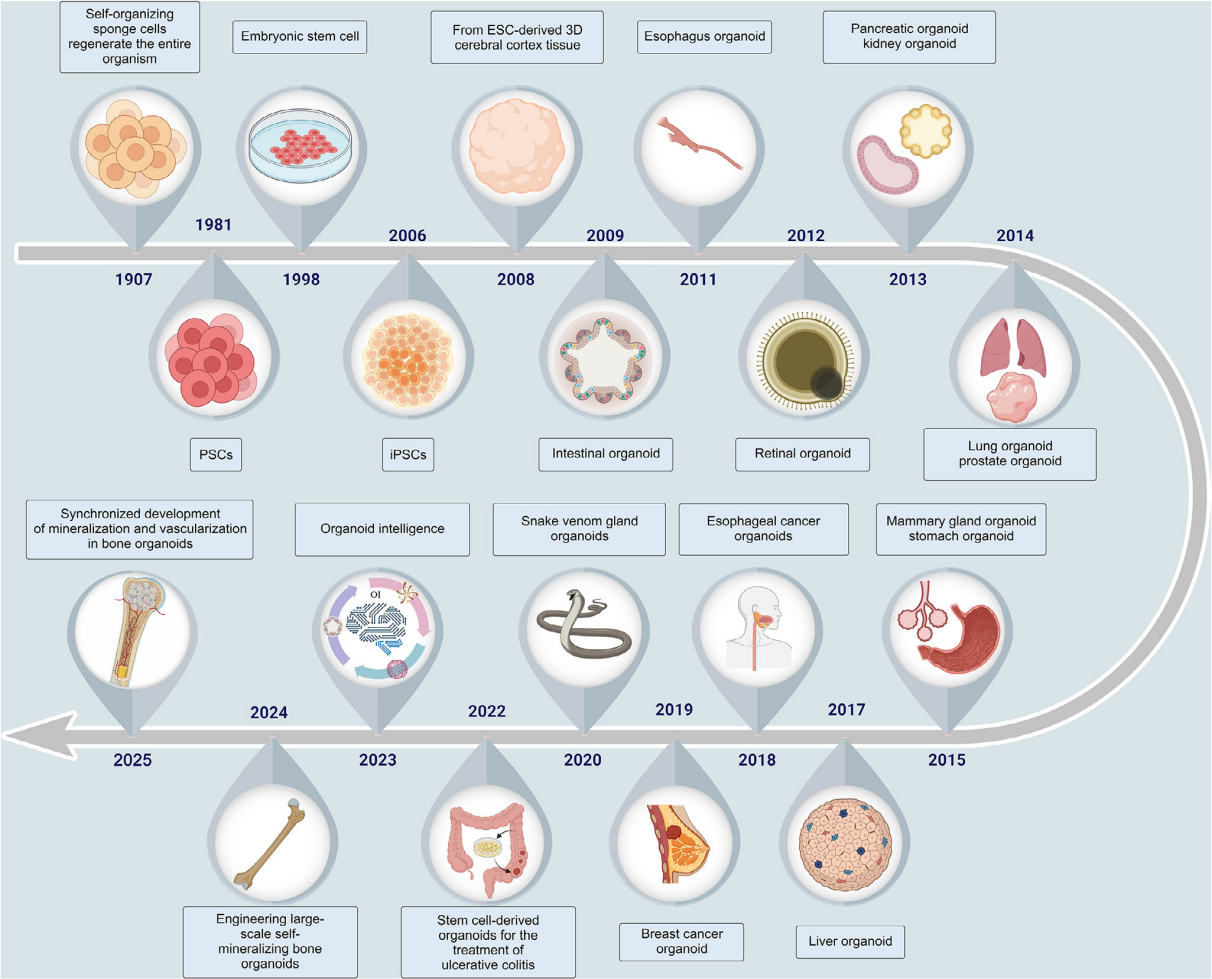
A brief history of organoids.

The past decade has witnessed significant milestones in organoid research (**Figure** **4**). In 2011, human intestinal and retinal organoids were first successfully cultured, establishing the feasibility of modeling diverse organ systems.^[^ ^24^ ^]^ The breakthrough construction of brain organoids by Lancaster and Knoblich in 2013 provided a powerful model for neurodevelopmental and neurological disease studies.^[^ ^25^ ^]^ Concurrent advances that year included successful cultivation of liver and kidney organoids, broadening the scope of organoid‐based disease modeling.^[^ ^26^ ^]^ Subsequent achievements in generating lung, prostate, and fallopian tube organoids further expanded their utility in respiratory and reproductive health research.^[^ ^27^ ^]^ Organoid technology extended beyond mammals in 2020, exemplified by the successful cultivation of venom gland organoids.^[^ ^28^ ^]^ A landmark clinical translation occurred in 2022, with Tokyo Medical and Dental University initiating the first transplantation of stem cell‐derived intestinal organoids into patients for ulcerative colitis, signifying a major advance towards regenerative therapies.^[^ ^29^ ^]^

Recent advances such as organoid intelligence (OI)—the integration of organoids with artificial intelligence (AI)—are opening new directions in biological computation. Meanwhile, combining organoids with organ‐on‐a‐chip technologies has enhanced the physiological relevance of 3D culture systems, improving disease modeling and drug screening accuracy.^[^ ^30^ ^]^ These innovations also reduce reliance on animal models and may increase clinical trial success rates.

### 3.2 Construction Strategies

**Figure 5:**
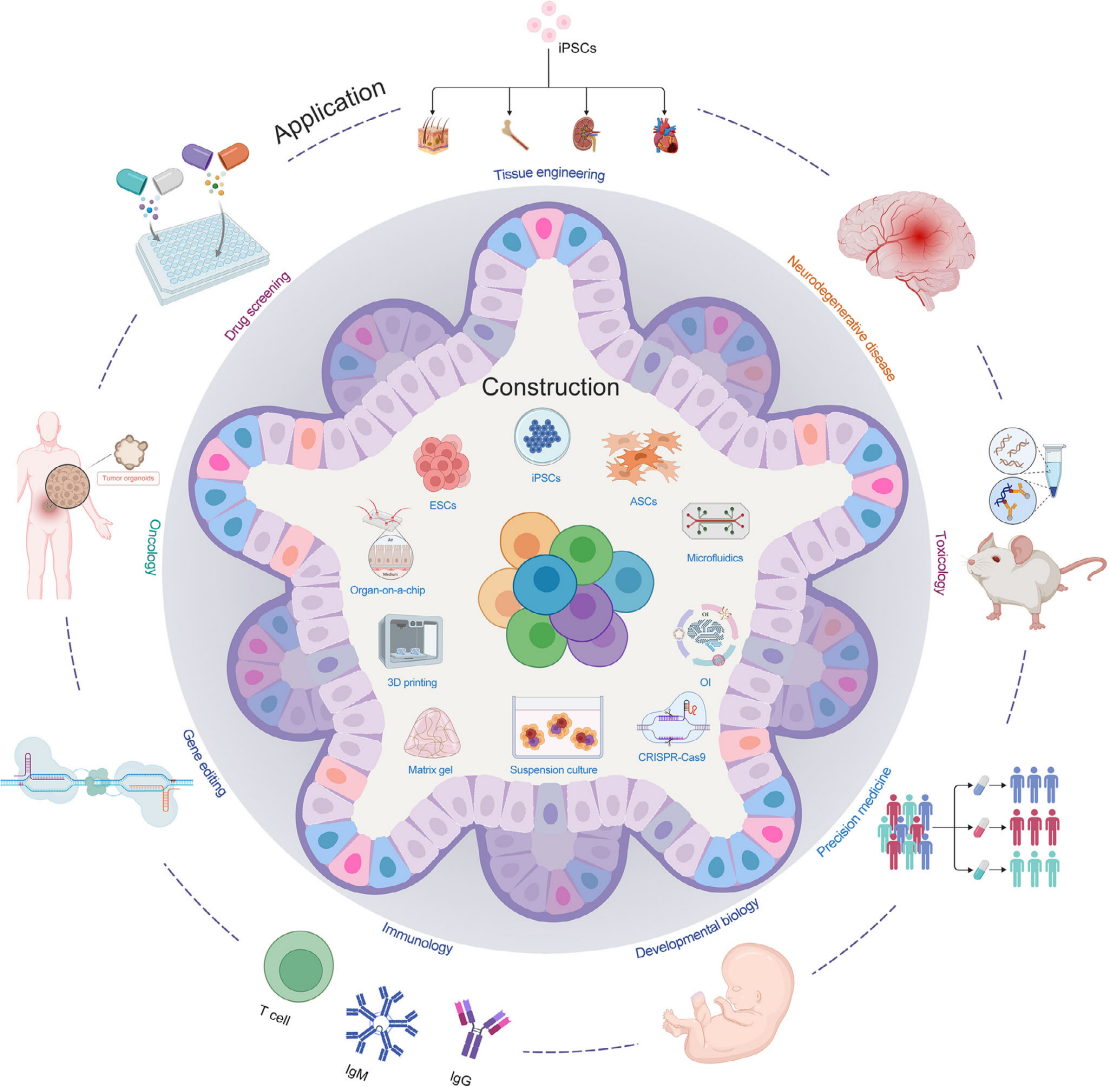
Construction and application of organoids.

Organoid construction techniques have evolved significantly through distinct developmental stages (**Figure** **5**). Initially, prior to 2010, embryonic stem cells (ESCs) and adult stem cells were primarily utilized due to their pluripotent differentiation capabilities. Notably, ESCs‐derived retinal organoids demonstrated early self‐organizing properties of complex tissues in vitro.^[^ ^24b^ ^]^

Between 2010 and 2014, methods such as matrigel embedding and suspension culture emerged prominently. Matrigel provided a supportive extracellular matrix analog crucial for organoid formation, exemplified by successful cultivation of intestinal organoids.^[^ ^31^ ^]^ Meanwhile, suspension culture facilitated the development of intricate structures such as brain organoids, enabling deeper exploration of tissue complexity and developmental biology.^[^ ^25^ ^]^ From 2015 to 2018, advancements in induced pluripotent stem cell (iPSC) technology and CRISPR‐Cas9 gene editing significantly enhanced organoid modeling precision. Patient‐derived iPSC organoids allowed personalized disease modeling and drug screening, while CRISPR‐Cas9 enabled targeted genetic manipulation to investigate disease mechanisms.^[^ ^32^ ^]^

Since 2020, organoid research has increasingly integrated microfluidic systems, organ‐on‐a‐chip, and AI, transitioning toward intelligent, dynamic, and highly functional models.^[^ ^30^, ^33^ ^]^ Microfluidics precisely replicate physiological environments through controlled nutrient exchange and dynamic flow, enhancing organoid maturity and functionality. Organoid‐on‐chip integration further refines drug screening efficacy and disease simulation accuracy.^[^ ^34^ ^]^ Concurrently, the convergence of organoid technology with AI has led to the innovative field of OI, transforming organoids into sophisticated biological computing units capable of elucidating complex biological processes.

### 3.3 Application Scenarios

Organoid technology has rapidly advanced, impacting diverse biomedical fields including oncology, neurodegeneration, drug screening, precision medicine, regenerative medicine, immunology, gene editing, and developmental biology (Figure 5).

#### 3.3.1 Oncology

Patient‐derived organoids (PDOs) faithfully replicate tumor heterogeneity and microenvironmental complexity, making them invaluable for cancer research. Breast cancer PDOs models effectively retain tumor‐infiltrating lymphocytes, illustrating critical immune checkpoint interactions, while co‐culture studies demonstrate organoids’ utility for T‐cell mediated therapies.^[^ ^35^ ^]^ Furthermore, organoids facilitate investigation into rare cancers, such as neuroendocrine prostate tumors, highlighting specific therapeutic targets like EZH2.^[^ ^36^ ^]^

#### 3.3.2 Neurodegenerative Diseases

Organoids represent a physiologically relevant platform for modeling neurodegenerative diseases, overcoming traditional limitations of animal and 2D models. Human brain organoids accurately reproduce hallmark features of Alzheimer's disease, such as amyloid plaques and tau tangles, enabling detailed study of disease progression and synaptic dysfunction.^[^ ^37^ ^]^ Similarly, patient‐derived organoids spontaneously exhibit dopaminergic neuron loss, providing insights into Parkinson's disease pathology without reliance on external toxins or genetic modifications, significantly advancing therapeutic research.

#### 3.3.3 Drug Screening and Toxicology

Organoids offer enhanced accuracy in predicting drug metabolism and toxicity compared to conventional cell lines or animal models. Intestinal organoids effectively model drug absorption and chemotherapy‐induced toxicity, demonstrating high‐throughput screening capabilities. Liver organoids excel in detecting hepatotoxicity, accurately reflecting drug‐induced injury such as fibrosis and oxidative stress, surpassing traditional hepatocyte assays. Kidney organoids replicate drug excretion mechanisms and toxicity responses, allowing real‐time metabolic monitoring and facilitating development of nephroprotective strategies.^[^ ^38^ ^]^

#### 3.3.4 Precision Medicine

Organoids accurately mirror patient‐specific tumor morphology and genetic profiles, significantly advancing precision oncology.^[^ ^39^ ^]^ Drug sensitivity tests using PDOs enable tailored treatment plans, optimizing therapeutic outcomes. Organoids maintain clonal heterogeneity over extended cultures, proving valuable in personalized drug response predictions, particularly for cancers exhibiting high treatment resistance.^[^ ^40^ ^]^

#### 3.3.5 Regenerative Medicine and Tissue Engineering

Organoids hold substantial promise in regenerative medicine, particularly for generating transplantable functional tissues. Successful transplantation of intestinal organoids demonstrates stable functional integration in vivo, highlighting clinical potential.^[^ ^41^ ^]^ In addition, engineered vascularized cardiac organoids exhibit complex physiological features, including autonomous beating and enhanced nutrient exchange, underscoring their potential in organ regeneration therapies.^[^ ^42^ ^]^

#### 3.3.6 Immunology

Thymic organoids represent powerful tools for studying T‐cell development and immune disorders. These organoids replicate the thymus microenvironment, supporting T‐cell differentiation, including regulatory T cells, and enabling detailed exploration of thymic selection processes.^[^ ^43^ ^]^ Such platforms enhance our understanding of immune cell maturation, thymus functionality, and provide novel avenues for therapeutic exploration in immune‐related conditions.

#### 3.3.7 Developmental Biology

Organoid technology revolutionizes developmental biology research by recapitulating complex organogenesis processes in vitro. Kidney organoid models elucidate critical signaling interactions, such as the coordinated actions of Wnt and BMP pathways, during nephron formation and renal differentiation.^[^ ^44^ ^]^ Likewise, lung organoids have clarified mesenchymal‐epithelial interactions essential for alveolar regeneration, highlighting the regulatory roles of Notch and Wnt/Fgf pathways.^[^ ^45^ ^]^ These findings significantly advance the understanding of developmental mechanisms and potential regenerative strategies.

#### 3.3.8 Gene Editing

Combining CRISPR‐Cas9 technology with organoids facilitates precise genetic manipulation, elucidating complex gene functions within authentic tissue contexts.^[^ ^46^ ^]^ Adult stem cell‐derived organoids further enhance the study of cellular differentiation, fate determination, and tissue‐specific regulatory mechanisms.

### 3.4 Challenges

#### 3.4.1 Insufficient Tissue Complexity and Diversity

Although organoids can reconstruct certain 3D structures and partial functions of organs in vitro, they still fail to fully replicate the complex in vivo environment. The absence of vascular systems, neural networks, and immune systems in organoids limits their application in studies involving complex physiological processes and multi‐tissue interactions. In addition, during long‐term culture, organoid tissues often face issues such as oxygen deprivation and the accumulation of metabolic waste, leading to cellular dysfunction and degradation.^[^ ^47^ ^]^ Although research has attempted to partially address this issue by incorporating endothelial cells and other strategies, the functionality and long‐term stability of vascularized organoids still require improvement. In addition, organoids lack complete interactions with the in vivo tissue microenvironment, such as the absence of immune cells and stromal cells, which limits their application in immunological research.^[^ ^35^ ^]^

#### 3.4.2 Lack of Standardization and Reproducibility

Organoid cultures exhibit significant variability across different laboratories, influenced by factors such as cell source, culture matrices, and conditions, resulting in poor reproducibility.^[^ ^48^ ^]^ This variability not only impacts the reliability of research findings but also limits the application of organoids in large‐scale drug screening and preclinical studies. Achieving standardization in organoid construction to improve consistency and reliability is a critical direction for future technological development.

#### 3.4.3 Long‐Term Maintenance and Functional Maturity

While organoids can form 3D structures resembling those in vivo, their structure and function gradually deteriorate during extended culture. This issue is especially prominent in neural and liver organoids, which require extended development periods and often fail to reach full maturity in vitro. Such early decline limits the application of organoids in long‐term experiments.

#### 3.4.4 Ethical and Regulatory Issues

Although organoids help circumvent some ethical concerns associated with animal experiments, ethical debates persist regarding certain complex organoids, particularly brain organoids.^[^ ^49^ ^]^ The complexity and potential consciousness‐related issues of brain organoids have raised concerns among ethicists, presenting a challenge in conducting such research within ethical guidelines.^[^ ^50^ ^]^ In addition, the clinical application of organoids lacks a clear regulatory framework, and ensuring their safety and efficacy in clinical translation remains an essential issue for the future.

Although organoids recapitulate many structural and functional features of native tissues, they remain largely restricted to modeling single organs in isolation. This limitation constrains their ability to capture the coordinated interactions that shape physiology and disease across tissue boundaries. To address these challenges, assembloids have been developed by combining multiple organoid types or distinct cell populations, enabling the study of inter‐tissue communication, circuit integration, and complex pathological processes that cannot be resolved by organoids alone.

## 4 Assembloids

### 4.1 Development History

Assembloids emerged as researchers recognized the limitations of single‐organoid systems in modeling complex tissue interactions. Defined as integrated 3D constructs assembled from multiple organoids or cell types.^[^ ^51^ ^]^ The concept was first demonstrated by Birey et al. in 2017, who established cortical‐dorsal/ventral forebrain assembloids that facilitated neuronal connectivity between glutamatergic and GABAergic populations, marking a significant advancement in modeling inter‐tissue interactions.^[^ ^4a^ ^]^

**Figure 6:**
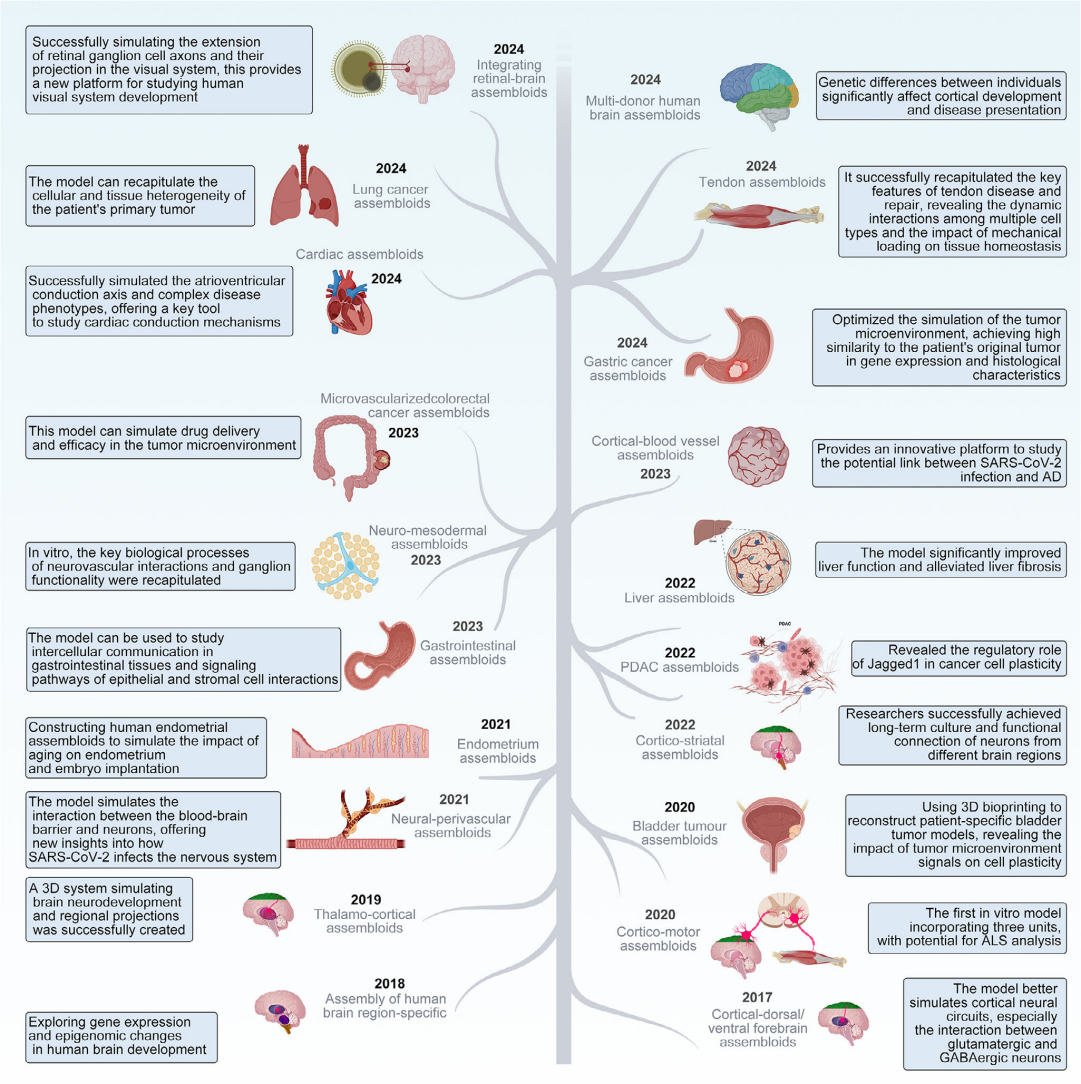
A brief history of assembloids.

Subsequently, assembloids applications rapidly expanded into neuroscience, oncology, and regenerative medicine, encompassing diverse tissues including brain, intestine, heart, pancreas, retina, bladder, gastric cancer, and bone.^[^ ^42^, ^52^ ^]^ Notably, Kim et al. employed 3D bioprinting to integrate bladder tumor organoids with stromal and immune cells, successfully recapitulating the tumor microenvironment.^[^ ^53^ ^]^ Pașca and colleagues advanced neuromuscular modeling by connecting brain, spinal cord, and muscle organoids, significantly aiding studies on motor neuron diseases like amyotrophic lateral sclerosis.^[^ ^54^ ^]^ Furthermore, their 2022 work merged cortical and striatal organoids to explore neural connectivity and differentiation.^[^ ^55^ ^]^ Most recently, Arlotta's team developed reproducible multi‐donor cortical assembloids, enhancing reliability and translational potential for human brain research (**Figure** **6**).^[^ ^56^ ^]^

### 4.2 Construction Strategies

**Figure 7:**
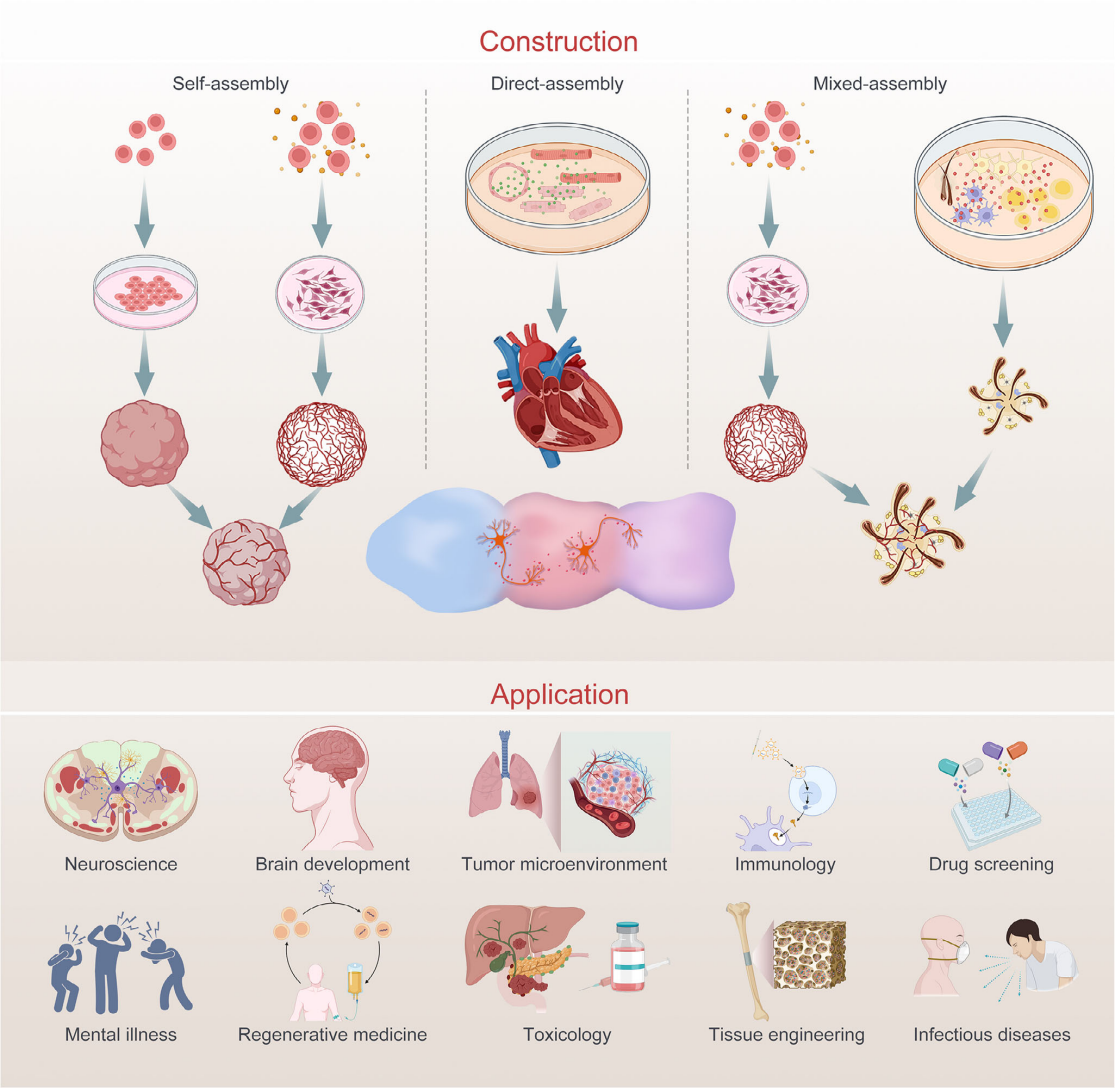
Construction and application of assembloids.

Current strategies for assembling assembloids include self‐assembly, direct assembly, and mixed assembly (**Figure** **7**), each offering distinct advantages in reconstructing the multicellular complexity of human tissues.

Self‐assembly, in particular, utilizes the intrinsic developmental potential of pluripotent stem cells to generate multicellular structures through spontaneous proliferation, aggregation, and fusion. By introducing specific cytokines into the culture medium, stem cells are directed to differentiate and generate supportive stromal lineages, such as endothelial and immune cells.^[^ ^57^ ^]^ This promotes the fusion of differentiated spheroids into coherent, functional tissue‐like assemblies.^[^ ^54^, ^58^ ^]^

For example, overexpression of ETS variant 2 (ETV2) in stem cells, combined with neural induction factors, facilitates the formation of vascularized cortical assembloids.^[^ ^59^ ^]^ Similarly, BDNF and NT3 support cortical neuronal maturation, while Smad inhibitors, retinoic acid, and Sonic Hedgehog (SHH) drive the differentiation of spinal motor neuron organoids. These components can be integrated with pre‐differentiated skeletal muscle cells to form neuromuscular assembloids capable of modeling motor circuits in vitro.^[^ ^54^ ^]^ Because self‐assembled assembloids originate from stem cells and encompass the entire developmental process of the cells, they hold significant advantages in the study of cell, tissue, and embryonic development, as well as in understanding the mechanisms of disease onset and progression.^[^ ^54^, ^58^, ^60^ ^]^

Direct assembly is a strategy that utilizes various differentiated and mature cells to form assembloids. Specifically, direct assembly involves co‐culturing pre‐isolated mature cells in appropriate ratios and methods, inducing them to fuse into a multicellular complex. For example, the Mills team differentiated stem cells in a culture medium containing bone morphogenetic protein 4 (BMP‐4), activin A, and vascular endothelial growth factor A (VEGFA), and then used flow cytometry to isolate cardiomyocytes and endothelial cells. These two cell types were co‐cultured in a 4∶1 ratio to assemble cardiac assembloids.^[^ ^61^ ^]^ Because direct assembly assembloids contain multiple differentiated and mature cell types with relatively well‐established functions, this model exhibits a higher degree of fidelity in simulating in vivo conditions.

Mixed assembly combines the advantages of both self‐assembly and direct assembly. The essential cells within the assembloids are derived from stem cell differentiation, while the co‐culture system includes mature stromal cells that possess some of the functions of in vivo stromal cells.

Mixed assembly refers to the process in which pluripotent stem cells, adult stem cells, and tumor stem cells proliferate and aggregate, followed by induced differentiation to form organoids or cell spheres. These organoids are then co‐cultured with various differentiated and mature cell types to create assembloids.^[^ ^52a^ ^]^ In recent years, this approach has been increasingly utilized to rebuild tumor microenvironments, further investigating the mechanisms of immune evasion within these environments with the aim of developing more effective immunotherapies.^[^ ^35b^ ^]^

### 4.3 Application Scenarios

Organoids are well‐suited for modeling the development and pathology of individual tissues, enabling precise investigation of molecular and cellular processes within a single organ system (Figure 7). In contrast, assembloids integrate multiple organoid types or cellular lineages to reconstruct inter‐organ interactions, thereby extending the capacity of traditional organoid systems to more accurately simulate the spatial and functional complexity of multi‐tissue environments. This allows for deeper exploration of complex diseases, immune crosstalk, regeneration, and pharmacological responses.

#### 4.3.1 Neuroscience and Brain Development

The formation of neural circuits is a critical process in brain development, involving the migration, connectivity, and functionalization of neurons. Assembloids create a multi‐region interactive 3D model by combining organoids from different brain regions, simulating how neurons communicate through complex signaling pathways. By utilizing organoids from various brain areas, such as the cortex, striatum, and midbrain, researchers can more accurately model the dynamic interactions between different regions of the brain, thus aiding in the exploration of brain function integration and the signaling abnormalities present in diseases.^[^ ^62^ ^]^

By linearly arranging midbrain‐striatal‐cortical organoids, Daniel Reumann et al. developed an in vitro assembloid model called MISCOs to simulate the human dopaminergic nervous system. This 3D structure mimics critical neural circuits in the human brain. MISCOs not only replicate the maturation process of dopaminergic neurons but also demonstrate how these neurons project to the striatum and cortex, forming functional neural connections.^[^ ^63^ ^]^

In addition, assembloids can be utilized to study the specialization of brain regions and the regulatory signaling pathways involved in brain development to simulate the signaling pathways during the process of brain regionalization, particularly highlighting the role of SHH signaling in the formation of different brain regions.

#### 4.3.2 Tumor Microenvironment and Immune Response

In cancer research, constructing models that accurately replicate the complex interactions between the tumor microenvironment (TMEs) and the immune system is crucial. Zhang et al. established patient‐specific lung cancer assembloids (LCAs), which effectively recapitulate heterogeneous TMEs, including immune cells and cancer‐associated fibroblasts.^[^ ^64^ ^]^ These LCAs successfully preserved the parental tumor cell composition, genomic characteristics, and functional heterogeneity.

#### 4.3.3 Research on Mental Disorders

The thalamus, acting as a central relay for sensory information, forms intricate bidirectional synaptic connections with the cortex. These connections play a crucial role in cognitive processes, emotional regulation, and behavioral control. In psychiatric research, functional abnormalities in cortico‐thalamic (CT) and thalamo‐cortical (TC) circuits are considered key pathological mechanisms underlying mental disorders, including schizophrenia, autism spectrum disorder, and bipolar disorder.

The TC assembloid serves as a powerful tool for exploring synaptic dysfunction in mental disorders by simulating the complex neural circuits and synaptic plasticity found in the human brain, particularly long‐term potentiation and long‐term depression.^[^ ^65^ ^]^ By three‐dimensionally assembling thalamic and cortical organoids, researchers can simulate the development and function of the CT and TC circuits. For instance, the assembloid model can aid in studying the abnormal connections in the TC circuitry of schizophrenia patients, revealing their role in reduced sleep spindle activity and impaired information processing.

#### 4.3.4 Regenerative Medicine and Tissue Engineering

Assembloids enhance the ability to simulate the structure and function of human tissues by integrating various types of organoids, cellular spheroids, or multicellular aggregates. Organ building blocks, functioning as multicellular units, can self‐assemble to form organ‐specific tissues that possess physiological functions, such as the vascularization and functional restoration of complex tissues like the heart, liver, and kidneys. By incorporating these assembloids into bioprinting and other tissue engineering methodologies, researchers have achieved highly complex engineered tissue structures, including vascularized cardiac tissue and functional liver tissue.^[^ ^66^ ^]^ Compared to organoids, assembloids offer a greater similarity to in vivo organs regarding cellular diversity and tissue heterogeneity, making them more promising for applications in organ regeneration.^[^ ^67^ ^]^

#### 4.3.5 Research on the Immune System and Infectious Diseases

Assembloids also exhibit significant potential in studying infectious diseases. Wang et al. developed a human 3D neural‐perivascular assembloid model incorporating pericyte‐like cells (PLCs) derived from human pluripotent stem cells to investigate the neuropathology of SARS‐CoV‐2 infection.^[^ ^68^ ^]^ This assembloid promoted astrocytic maturation and the formation of neurovascular unit structures. Critically, the presence of PLCs significantly enhanced neural susceptibility to SARS‐CoV‐2 infection, serving as viral replication hubs and facilitating subsequent spread to astrocytes. Following infection, astrocytes within the assembloid exhibited prominent inflammatory type I interferon responses and apoptosis.

#### 4.3.6 Drug Screening and Toxicology Applications

Compared to conventional 2D culture systems, assembloids incorporate functional architectures of multiple organs and more closely recapitulate physiological states observed in clinical settings.^[^ ^64^ ^]^ By simulating complex inter‐organ metabolic pathways, they offer improved accuracy in predicting patient‐specific drug responses and enhance the precision of drug toxicity screening.

### 4.4 Challenges

#### 4.4.1 Limitations in Tissue Complexity and Multi‐organ Interactions

Current assembloid models often only combine a limited number of organ or tissue types, making it challenging to fully replicate the intricate interactions between multiple organs in vivo. Although assembloids can effectively simulate interactions within the nervous system, such as inter‐regional communication in the brain or neuro‐immune system interactions, they frequently lack the resolution and functionality needed to replicate organ‐level interactions, such as those involving the nervous, vascular, and immune systems concurrently participating in complex physiological functions.^[^ ^4^, ^69^ ^]^

#### 4.4.2 Insufficient Vascularization and Microenvironment Support

Similar to organoids, assembloids also face challenges related to inadequate vascularization. Without proper vascular support, tissue growth is limited, and long‐term culture is difficult to maintain function, especially in studies of tissue repair and regeneration. Vascularized assembloid models still experience limitations in functional maturity. Although the introduction of endothelial cells can simulate angiogenesis, these microvascular networks often fail to fully replicate the functions of in vivo vasculature, such as blood flow regulation, signaling, and dynamic responses. Furthermore, factors such as intercellular communication, matrix components, and mechanical stress present in the in vivo microenvironment are difficult to fully replicate in assembloids. These limitations restrict their ability to accurately reflect real physiological processes.

#### 4.4.3 Standardization and Reproducibility Issues

The construction of assembloids typically relies on advanced spatial control techniques such as acoustic tweezers or 3D bioprinting. Acoustic tweezers offer the advantage of non‐invasive manipulation of cells; however, factors like acoustic wave intensity, frequency, and the selection of cell types can affect the stability of experimental results, leading to inconsistencies between different laboratories.^[^ ^70^ ^]^ In addition, the heterogeneity within assembloids is a crucial factor affecting reproducibility. Different combinations of organoids and cell types may exhibit inconsistent growth patterns in actual applications due to variations in the microenvironment and nutrient supply.^[^ ^71^ ^]^ Moreover, the heterogeneity of the internal microenvironment of assembloids poses another challenge to standardization. Even under identical culture conditions, variations in oxygen and nutrient distribution within the assembloids can lead to heterogeneity in cell growth and differentiation, affecting the predictability of outcomes. This microenvironmental variability complicates the assurance of stability and reproducibility across different experiments over extended culture periods.

#### 4.4.4 Tissue Degeneration during Long‐Term Culture

Assembloids are susceptible to issues related to inadequate nutrient and oxygen supply, particularly those that lack vascularization. Insufficient nutrient supply can lead to cell necrosis and apoptosis in the central regions of organoids, adversely affecting the overall health and functionality of the tissue. In addition, long‐term cultures may exhibit cell heterogeneity and instability in tissue structure, leading to inconsistencies in experimental results. In the striatum‐nigral assembloids model for Huntington's disease, researchers have observed that neuronal projection functions perform well in the early stages; however, as culture duration extends, the functionality of medium spiny neurons gradually declines, revealing significant signs of neurodegeneration. This decline has been associated with microenvironmental heterogeneity within the assembloid, where limited oxygen and nutrient diffusion generates spatial disparities in cell viability and maturation. Peripheral regions typically retain healthier neuronal activity, whereas central zones are prone to hypoxia, metabolic stress, and progressive loss of neuronal stability, thereby accelerating functional deterioration.^[^ ^72^ ^]^

#### 4.4.5 Technical Complexity and High Costs

The technical complexity and high costs associated with assembloids represent significant barriers to their current application and broader adoption. First, the construction of assembloids typically involves the integration of multiple high‐precision technologies, such as 3D bioprinting, microvascular design, and the directed differentiation of stem cells. These techniques require advanced equipment, skilled personnel, and well‐equipped laboratory infrastructure, making them less accessible to many research groups. Second, maintaining the growth and functionality of assembloids necessitates the use of expensive culture media, specific biomaterials, and sophisticated dynamic culture systems.^[^ ^71^ ^]^ The financial burden of these resources can be substantial, particularly for laboratories with limited funding.

##### 4.4.5.1 Ethical Considerations Unique to Assembloids

Recent advances in assembloid technology raise a series of ethical considerations that extend beyond those associated with parabiosis and brain organoids. Unlike single‐tissue organoids, assembloids involve the integration of distinct neural regions or tissues from different donors, which introduces unique concerns.^[^ ^62^, ^73^ ^]^ First, the increasing complexity of neural integration within assembloids raises questions about the potential emergence of higher‐order functions, including primitive network‐level activity that could be associated with consciousness‐like states. Although current evidence indicates that such capacities remain remote, their theoretical possibility requires continuous ethical vigilance. Second, the use of tissues or cells from multiple human donors introduces issues of consent, identity, and ownership, particularly when genetic backgrounds are combined in a single construct. Finally, as assembloids are increasingly applied in neurodevelopmental and neuropsychiatric disease modeling, the prospect of generating models that partially replicate cognitive or affective functions compels careful consideration of the boundaries of moral status and the potential for sentience. Addressing these concerns will require proactive guidelines tailored to assembloids, complementing existing frameworks for organoid and animal research.

## 5 Outlook

Future research in parabiosis should prioritize the precise identification and functional analysis of systemic blood factors, focusing on their roles in aging, immune regulation, and tissue repair. Advances in noninvasive blood modulation techniques will increasingly replace traditional invasive procedures, allowing safer clinical translation. Integration with stem cell therapies, organ‐on‐a‐chip systems, and microfluidic technologies will facilitate precise control and simulation of blood factor activities in vitro, reducing animal use and improving experimental reproducibility. Personalized blood‐factor therapies developed through detailed molecular profiling will enable tailored treatment strategies, enhancing patient‐specific clinical outcomes.

Organoids’ future advancements hinge upon achieving standardization, scalability, and enhanced functional complexity. Establishing rigorous culture protocols and quality control standards will be critical for widespread clinical and industrial applications.^[^ ^22^, ^74^ ^]^ Incorporation of vascularization and dynamic culture systems will substantially increase their physiological accuracy and functional maturity.^[^ ^75^ ^]^ Recent progress in organoid research has included the establishment of dedicated biobanks and the development of standardized culture kits. Large‐scale biobanks now collect patient‐derived organoids from multiple tissues and diseases, together with molecular and pharmacological reference data that enable reproducibility across laboratories. In addition, commercially available kits for intestinal and other patient‐derived organoids provide defined media formulations and uniform protocols that support consistent culture and large‐scale expansion. These initiatives have begun to provide the practical framework required to address issues of heterogeneity and scalability in translational applications.^[^ ^76^ ^]^ Organs generated from organoids not only have the potential for use in in vitro drug screening but also offer new solutions for tissue repair and regeneration, with the potential to treat patients with various organ dysfunctions. In the future, researchers are expected to develop personalized organ transplantation strategies using organoid technology, particularly for the reconstruction of functional organs such as the liver and kidneys.^[^ ^77^ ^]^

The combination of organoid technology with organ‐on‐a‐chip platforms will further refine simulation of dynamic physiological environments, significantly improving drug screening accuracy and reducing reliance on animal models. Precision gene editing approaches, especially CRISPR‐Cas9, will facilitate the detailed investigation of genetic disease mechanisms and therapeutic interventions.^[^ ^75^, ^78^ ^]^ Furthermore, the integration of AI‐driven analytics will enable efficient processing of complex datasets from organoid studies, propelling personalized medicine by accurately predicting individual therapeutic responses.^[^ ^79^ ^]^

For assembloids, future efforts will center on replicating dynamic multi‐organ interactions and complex physiological functions, particularly emphasizing systems like the heart‐brain and gut‐brain axes. Enhanced incorporation of diverse cell types, such as neurons, endothelial cells, and immune components, will significantly improve the physiological relevance and functional fidelity of assembloids. Advances in vascularization methods and microfabrication techniques will address current limitations, allowing assembloids to more accurately mimic real‐time blood flow dynamics and tissue microenvironments.^[^ ^53^ ^]^ A dynamic microenvironment can significantly aid cell behaviors such as migration and axon projection. Simulating dynamic microenvironmental variables‐such as fluid shear stresses, oxygen, and nutrient gradients‐shall better mimic in vivo tissue behaviors, increasing the functionality and stability of assembloids.

With the advances of gene editing tools like CRISPR, assembloids will enable larger‐scale high‐throughput screening, unveiling intricate disease mechanisms, and enable higher‐precision drug screening. In addition, the structural diversity and complexity of assembloids make them better systems of gene interaction studies, offering higher utility within the analysis of gene function and drug target validation.^[^ ^53^, ^80^ ^]^ The combination of assembloids with gene editing and biomaterial science in repairing complicated tissue damage and constructing organ transplant materials will continue to propel the field of autologous tissue repair and organ regeneration, allowing for replacement tissues that are closer to patients’ physiological states. In addition, assembloid models derived from patients’ tumor cells have successfully recreated the tumor microenvironment, enabling personalized drug screening and efficacy prediction. In the future, with progress in high‐throughput gene screening and assembloid models, physicians will be able to design more precise, individualized treatment plans, enhancing therapeutic outcomes and minimizing side effects.^[^ ^64^ ^]^ Soft robotics technology can significantly enhance assembloids by introducing dynamic mechanical stimulation and real‐time controllability, better replicating physiological tissue interactions. Integrating soft robotic actuators within assembloid systems could simulate organ‐specific biomechanics, such as cardiac rhythmic contractions or peristaltic movements in gastrointestinal tissues.^[^ ^81^ ^]^ This approach provides precise regulation of mechanical forces and tissue deformation, promoting maturation and functional fidelity of assembloids.

**Figure 8:**
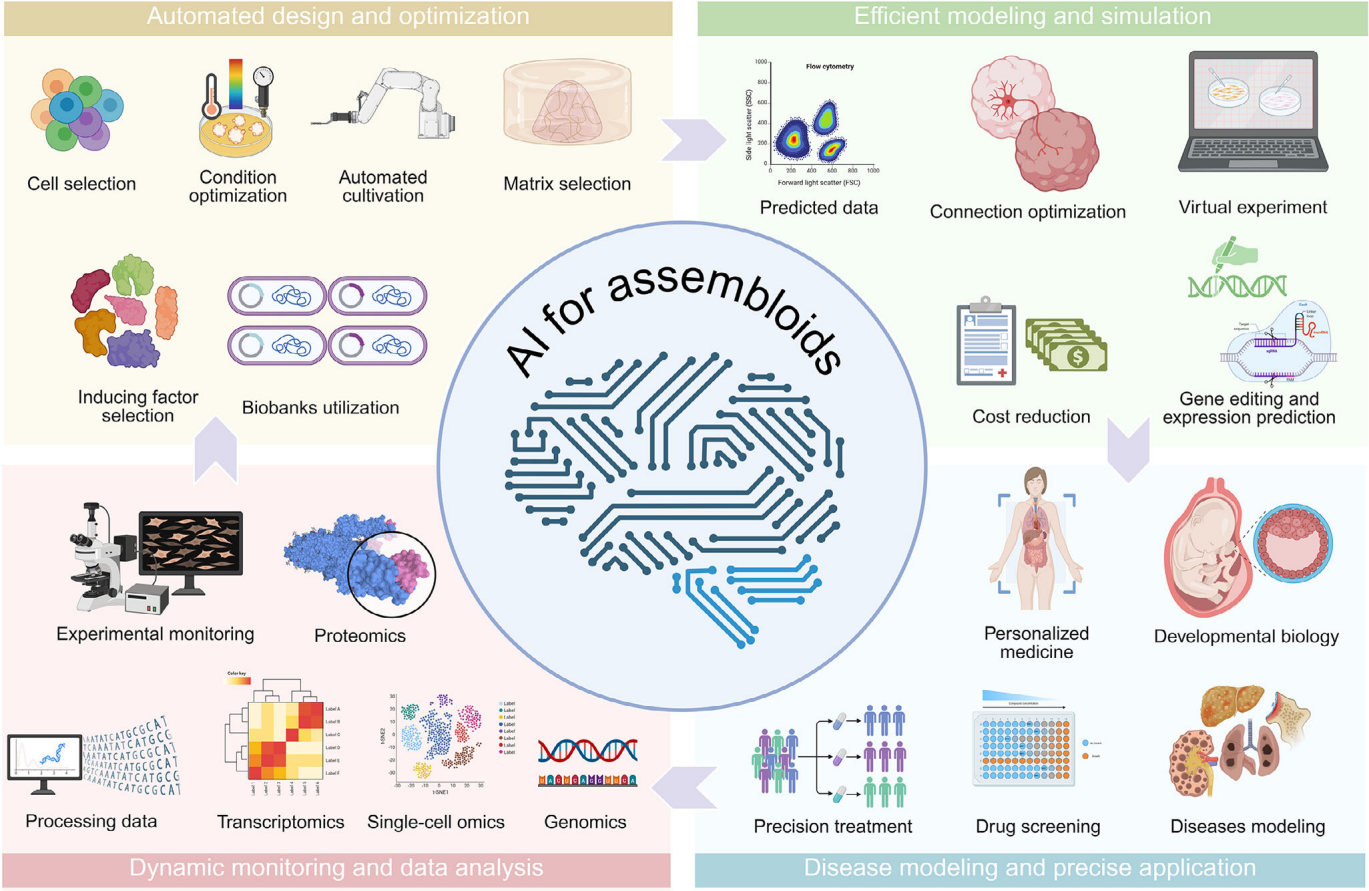
The potential applications of AI in assembloids.

With the current research findings, the combination of assembloids and machine learning/AI is likely to greatly spur the advancement of the biomedical field. Through the examination of multidimensional data from assembloids—gene expression, cell behavior, and tissue formation—AI and machine learning can quickly pick out important attributes related to the development of a disease and the response of drugs. In drug screening and individualized medicine, the machine learning/AI model can identify potential targets of drugs from high‐throughput screening information produced by the assembloids and predict the efficacy and toxicities of the drugs toward various patients. This can greatly speed up the workflow of producing a new medicine. Furthermore, AI can aid in analyzing the growth and differentiation patterns of assembloids under various experimental conditions, allowing for the optimization of culture conditions and enhancing the reproducibility and stability of experiments (**Figure** **8**).

In addition, integrating AI with assembloids will drive more precise disease modeling and mechanistic research. Through deep learning on assembloid data, AI can uncover the developmental and pathological changes of complex tissues, providing more accurate models to simulate in vivo environments. This combination will offer powerful tools for future biomedical research, advancing the application of assembloids in disease diagnosis, therapeutic strategy development, and tissue engineering to new levels.^[^ ^68^ ^]^

The rise of PAO reflects a profound transformation in biomedical research, particularly in the exploration of disease mechanisms and the development of therapeutic strategies. Successful translation of organoid and assembloid systems faces challenges in reproducibility, standardization, and regulatory alignment. Emerging collaborative networks emphasize shared biobanks and harmonized protocols, underscoring that future progress will rely on coordinated efforts between academic, industrial, and regulatory communities. These three model systems reshape the way biological systems are simulated from different dimensions, providing powerful tools at various levels for understanding disease complexity, accelerating drug discovery, and advancing regenerative medicine. Parabiosis emphasizes the interaction of systemic factors, revealing how circulating factors affect physiological functions and pathological processes across tissues. In studies of aging, metabolic disorders, and immune system function, parabiosis has demonstrated its unique advantages.

In contrast, organoids and assembloids focus on more refined reconstructions at the organ and tissue levels, providing biologically meaningful in vitro models for a deeper understanding of disease manifestation in terms of tissue structure and function.^[^ ^82^ ^]^ Organoids replicate the structure and function of individual organs, making them highly effective for studying organ development, disease progression, and drug responses. Assembloids, on the other hand, integrate different types of organoids or cell populations to form complex multi‐organ systems, addressing the limitations of single‐tissue models.^[^ ^51^ ^]^ This enables more accurate simulation of complex multicellular biological processes such as inter‐organ interactions, neural circuit connections, and immune system responses.^[^ ^73^ ^]^ Together, PAO provide a continuum of models that span systemic, organ, and multi‐organ levels, enabling comprehensive investigation of disease pathophysiology and supporting the advancement of precision medicine.

In the future, the integration of these three model systems is expected to open up new applications in the biomedical field. With the advancement of microfluidic and multi‐organ chip technologies, organoids and assembloids will not only operate in more dynamic and refined in vitro environments, but also significantly enhance the effectiveness and safety of drug screening by simulating the human microenvironment. The systemic factors revealed by parabiosis will provide clearer molecular targets and mechanisms of action for organoids and assembloids. As the in vivo microenvironment is more accurately replicated and biological factors are precisely controlled, these models will accelerate the development of innovative therapies, personalized treatments, and disease prevention strategies. Recently, the U.S. Food and Drug Administration issued a statement on its official website, marking a groundbreaking step toward improving public health: it will gradually move away from animal testing in the development of monoclonal antibodies and other drugs, shifting instead toward “more effective, human‐relevant research methods.” This transition presents a historic opportunity for the advancement of organoids and assembloids.

The 2024 Nobel Prize in Physics was awarded to John J. Hopfield and Geoffrey E. Hinton in recognition of their pioneering contributions to artificial neural networks and machine learning, underscoring the transformative impact of AI on interdisciplinary scientific progress. Building upon this momentum, the integration of AI with organoid technology is catalyzing a new phase of intelligent innovation.^[^ ^83^ ^]^ In particular, machine learning algorithms are redefining approaches to scientific inquiry by enabling more efficient construction, precise evaluation, and translational application of organoids and assembloids.^[^ ^84^ ^]^

Therefore, the complementary and synergistic integration of PAO will advance human understanding and intervention in health at a higher level. Through an integrated research framework that spans from the whole system to the microscopic level, future biomedical research and clinical practice will enter a new era of precision, intelligence, and personalization. This shift will significantly shorten the time from basic research to clinical application, empowering humanity with greater capabilities to combat diseases and extend healthy lifespans.

## Conflict of Interest

The authors declare no conflict of interest.

## Data Availability

I agree that if accepted, the article will be published open access and that the Corresponding Author is responsible for arranging payment of the APC.
